# Flexible Kernel Memory

**DOI:** 10.1371/journal.pone.0010955

**Published:** 2010-06-11

**Authors:** Dimitri Nowicki, Hava Siegelmann

**Affiliations:** 1 Biologically Inspired Neural and Dynamical Systems (BINDS) Lab, Department of Computer Science, University of Massachusetts Amherst, Amherst, Massachusetts, United States of America; 2 Institute of Mathematical Machines and Systems Problems of Ukraine National Academy of Science (IMMSP NASU), Center for Cybernetics, Kiev, Ukraine; 3 Program on Evolutionary Dynamics, Harvard University, Cambridge, Massachusetts, United States of America; Cuban Neuroscience Center, Cuba

## Abstract

This paper introduces a new model of associative memory, capable of both binary and continuous-valued inputs. Based on kernel theory, the memory model is on one hand a generalization of Radial Basis Function networks and, on the other, is in feature space, analogous to a Hopfield network. Attractors can be added, deleted, and updated on-line simply, without harming existing memories, and the number of attractors is independent of input dimension. Input vectors do not have to adhere to a fixed or bounded dimensionality; they can increase and decrease it without relearning previous memories. A memory consolidation process enables the network to generalize concepts and form clusters of input data, which outperforms many unsupervised clustering techniques; this process is demonstrated on handwritten digits from MNIST. Another process, reminiscent of memory reconsolidation is introduced, in which existing memories are refreshed and tuned with new inputs; this process is demonstrated on series of morphed faces.

## Introduction

Memory experiments demonstrated persistent activity in several structures in the lower mammal, primate, and human brains including the hippocampus [1], [2], prefrontal [3], visual [4] and oculomotor cortex [5], basal ganglia [6], etc. Persistent dynamics is believed to emerge as attractor dynamics (see also [7]–[13]). Currently, the leading paradigm in attractor neural networks memory models is the Hopfield model [14] with possible variations, including activation functions, neural firing, density of the neural connections, and the memory loading paradigms [15]–[22].

In this paper, we introduce a memory model, in which its memory attractors do not lie in the input or neural space as in classical models but rather in a feature space with large or infinite dimension. This model is isomorphic to a symmetric Hopfield network in the kernels' 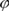-space, giving rise to a Lyapunov function in the dynamics of associative recalls, which enables the analogy to be drawn between memories and attractors in the kernel space.

There are several advantages to our novel kernel approach to attractor memory. The input space can be composed of either continuous-valued or binary vectors. The number of attractors 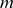 is independent of the input dimension 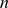, thus posing a saturated-free model, which does not suffer corrupted memories with memory overload. Attractors can be efficiently loaded, deleted, and updated on-line, something that has previously been only a property of symbolic computer-memory models. Furthermore, for the first time in neural memory models, we have demonstrated a method allowing input dimension not to be constrained to a fixed size or be a priori bounded; dimension can change with time, similar to organic memory allocation for memories of greater importance or increased detail. These attributes may be very beneficial in psychological modeling.

The process of kernel memory consolidation results in attractors in feature space and Voronoi-like diagrams that can be projected efficiently to the input space. The process can also describe clusters, which enables the separation of even very close-by attractors. Another re-consolidation process enables tracking monotonic updates in inputs including moving and changing objects.

### Generalizing Radial-Basis-Function Networks

Our network can be thought of as generalizing Radial Basis Function (RBF) networks [23]. These are 2-layered feed-forward networks with the first layer of neurons having linear activation and the second layer consisting of neurons with RBF activation function. Recurrent versions of the RBF networks [24], [25] add time-delayed feedback from the second to the first layer. Our network enables a more generalized structure, both in terms of number of layers and in allowing for many more general activation functions.

Unlike previous RBF networks, our activation functions are chosen from a large variety of kernels that allow us to distinguish attractors that are similar or highly correlated. Furthermore, unlike any previous RBF network with its fixed architecture and activation functions, our selected neural kernel functions can change during learning to reflect the memory model's changing properties, dimension, or focus. We go on to prove that the attractors are either fixed points or 2-cycles, unlike general recurrent RBF networks that may have arbitrary chaotic attractors [26], [27]; regular attractors are advantageous for a memory system.

### Synaptic plasticity and Memory Reconsolidation

Reconsolidation is a process occurring when memory becomes liable during retrieval and can then be updated. This process is implicated in learning and flexible memories when healthy; it leads to amnesia and compulsive disorders when corrupted. Reconsolidation is observed both in neurophysiological and psychological studies ([28]–[33]) and has been modeled in artificial neural systems as well ([19], [34]). While the actual processes underlying reconsolidation are still being studied, the property of dependance on sample ordering has been established in both electrophysiology of CA3 neurons [13] and in psychophysics [35]. In reconsolidation, memory representations are sensitive to the order of sample data: when samples change in an orderly manner, the reconsolidation process learns and updates effectively. When samples are shuffled and consistent direction of change is lost, existing memories do not update. We show here that the importance of input ordering is inherent in any update processes, reminiscent of reconsolidation. We also demonstrate how reconsolidation works in flexible environments and with large-scale data beyond the model shown in [19].

Our flexible model assumes global memory update. This is an interesting approach for a few reasons. First, it results in more stable and robust updates: in other models the “closest” attractor may be selected incorrectlyly due to noise. Second, it enables a direct analogy to an existing neural model of reconsolidation [19] since there the whole synaptic matrix is adjusted, not simply a chosen attractor. Moreover with global updates our memory can demonstrate phenomena analogous to the gang effect [36]. While we have taken a global update approach our model retains the property in which the retrieved attractor (the attractor closest to the current input) is most affected.

### Kernel Based Algorithms and Memories

The memory system introduced here takes advantage of developments introduced in Support Vector Machine (SVM) [37], Least-Square SVM [38] and Support Vector Clustering [39], where kernel functions enable data handling in higher feature spaces for richer boundaries, yet do so efficiently and cheaply. Our support-vector-like memory system incorporates the realistic property of flexible attractors with high dimensional feature spaces, while being tractable and implementable by neural units.

Zhang et al. [40] introduced a feedforward network with particular kernels in the form of wavelet and Radial Basis Functions (RBF) that were fit to perform a face recognition task efficiently. The kernel heteroassociative memories were organized into the modular network. Our architecture can be recurrent, which is more powerful than the feedforward method, can handle discrete and analog inputs, and the kernels we use can change online adding increased flexibility and capability.

Caputo [41] explored analogies from associative memory to “kernel spin glass” and demonstrated an attractor network, loading bipolar inputs and using generalized Hebbian learning to load non-flexible memories with greater memory capacity than the Hopfield network. In this work, a kernel algorithm generalized the Hebbian rule and the energy function of Hopfield networks, while capacity estimations generalized Amit's approach [1]. This method built in the free energy function in addition to the Hamiltonian. Our system, by comparison, allows for both binary and continuous inputs, is far more flexible in that the kernels adapt themselves over time and that attractors and features can be added and removed. Further, our system is more practical in that it has the added capability to cluster data.

Support vector memory by Casali et al. [42] utilized support vectors to find the optimal symmetric Hopfield-like matrix for a set of binary input vectors. Their approach is very different from ours despite the similar title, in that it considers only binary symmetric case and has bounded attraction space. Support-vector optimization is used to find optimal matrix 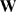 for given 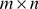 matrix 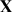 of etalons. This matrix must satisfy relationship 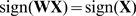. Support vectors are found to provide optimal margins of 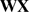. Kernels are not used in this work and hence the name is somewhat confusing. Our kernel memory is far richer: the number of memory attractors is not bounded by input dimension - accomplished by varying the input space; our encoding is more efficient, our memory can use discrete or analog space, one-shot learning, and overall is more flexible.

In support vector clustering [39], clusters are formed when a sphere in the 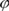-space spanned by the kernels is projected efficiently to input space. Here the clustering is a side effect of the created memories that are formed as separated fixed points in the 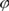-space, and where the Voronoi polyhedron is projected on a formation of clusters in the input space. Formation of memories is local, sharing this concept with the Localist Attractor Network [43] and the Reconsolidation Attractor Network [34].

### Organization

This work will be presented as follows: At first, the model of kernel heteroassociative memory is introduced, followed by the special case of auto-associative memory where attractors emerge. A neural representation is layered, and robustness (attraction radius) is estimated. We then introduce a technique that allows adding and removing attractors to the existing kernel associative network, and follow by introducing another technique that adds or removes input dimensionality on line. We next show a procedure of consolidating data into representing attractors, and demonstrate clusters emerging on handwritten digits and conclude by introducing the functional level of reconsolidation in memory and applications to morphed faces.

## Results and Discussion

### 2.1 Our Kernel Heteroassociative Memory Model

A general framework of heteroassociative memory can be defined on the input 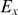 and output 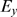 spaces, with dimensionalities 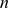 and 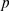 respectively, and with 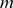 pairs of vectors 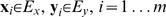 to be stored. The input vectors in the space 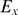 can be written as columns of matrix **X** (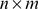) and associated vectors in the output space 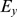 as columns of matrix **Y** (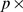
m). A projective operator 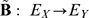 such that 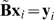 can be written in a matrix form 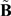 with
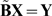
(1)and be solved as
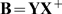
(2)with “
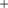
” stands for the Moore-Penrose pseudoinverse of 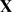 [44]. If the columns of 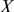 are linearly independent, the pseudoinverse matrix can be calculated by
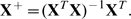
(3)


Let us define matrix 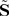, (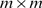), where the elements 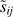 are the pairwise scalar products of the memorized vectors, that is 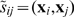, or in matrix notation:
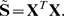
Then 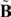 can be written as:
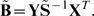
(4)We propose to formulate the pseudoinverse memory association (recall) by calculating for each input vector 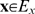 the output by:
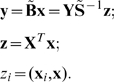
(5)This is a “one-pass” non-iterative linear associative memory. It has the property that if two input samples are close to each other, then the two outputs will be close to each other as well: 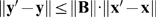.

#### 2.1.1 Memory in Feature Space

In order to overcome the common dependence of memory capacity on input dimension, we transform the input space 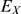 to a new input space 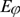 which we call feature space, whose dimensionality can be far greater than the dimension of 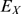, 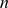 (it could even be an infinite-dimensional Hilbert space). The transformation 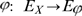 is considered to be transferring from input to feature space.

The respective associative memory algorithm can now be defined as follows:
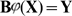
(6)

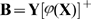
(7)

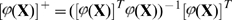
(8)Analogously writing 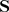 as
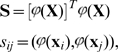
(9)the memory loads by:
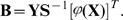
(10)and the association (recall) procedure is calculated by:
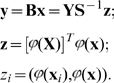
(11)


**Remark 1** Linear independence of the vectors 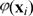 in the 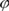-space is required in order to use identity (3) for the Moore-Penrose pseudoinverse (see [44]). It is achieved as we will see below by using piece-wise Mercer kernels, and does not limit the number of attractors. This identity is used to bring equation (7) to the form of (8) and to introduce 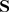.

We note that during both loading (10) and recall (11) procedures, the function 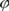 appears in the pair 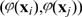. We can thus define a Kernel function over 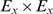 and gain computational advantage.

Let us denote a scalar product in the *feature space* 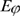 by 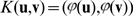. This is a symmetric, real-valued, and nonnegative-definite function over 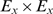 called a *kernel* [37]. We now can write 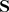 and 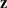 using the Kernel 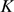:
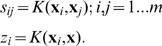
(12)The value of the Kernel function is scalar. Thus even if 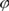 was a function of high dimension the calculation of the multiplication is a scalar and thus fast to calculate.

Mercer kernels as used in Support Vector Machines [37] are not sufficient for creating the associative memory we introduce, since our memories also require that all attractors are linearly independent in the feature space. To enable such independence we define the piece-wise Mercer kernels and in Section “Piece-wise Mercer Kernels” of Materials and Methods (MM) we prove that they can always be found and always lead to independence.

**Figure 1:**
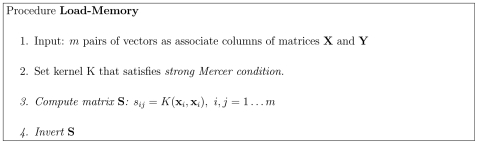
The algorithm of memory loading.

As opposed to Hebbian learning that requires O(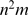) multiplications, we need 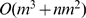 arithmetic operations over real scalars. The loading algorithm is displayed in Fig. 1. The memory is proven below to associate loaded pairs correctly and to associate close by values otherwise (see Materials and Methods (MM)).

#### 2.1.2 Memory Independent on Input Dimension

The kernel heteroassociative memory has no a priori bound on capacity in the following sense: for any given learning sample there exists a kernel such that the memory with this kernel will provide the desired association.

To specify this we formulate the following theorem, which is proven in MM Section “Correctness of Association”:

**Theorem 1** *For any memory size* 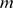, *let* 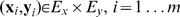 *be a learning sample consisting of* 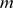 *input-output pairs. Then there exists a piece-wise Mercer kernel* 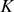 *such that the associative memory that has this kernel and governed by* *equations (9)* *–* *(11)* *assigns* 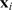 *to* 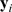 *for all* 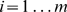.

**Remark 2** For the correct association, the memories have to be linearly independent in the feature space. As we have shown here this does not pose a memory limit, because for any given learning sample we can find a (piece-wise Mercer) kernel that guarantees such independence.

### 2.2 The Kernel Autoassociative Memory Model

We next focus on the special case where 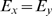, and the stored vectors 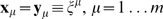. Here the loading algorithm is the same as in Fig. 1, and recall is facilitated by the iterative form:
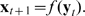
(13)The activation function 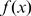 is applied by coordinates and constitutes a bounded monotonically increasing real-valued function over 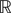 such that 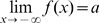, and 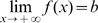, 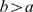.

**Figure 2:**
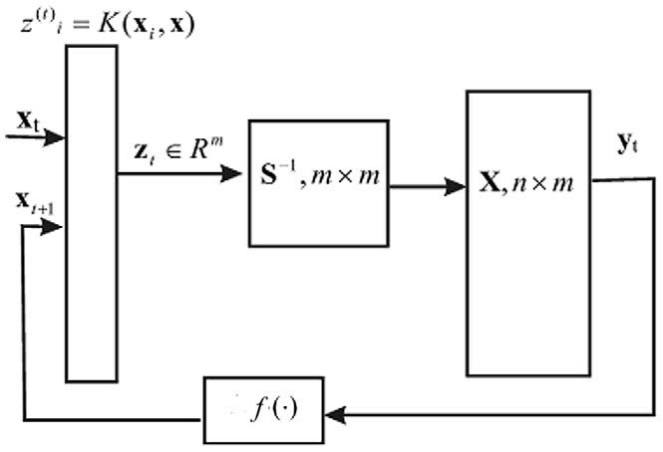
Scheme of the kernel autoassociative memory.

The scheme of kernel auto-associative memory working in recall mode is shown in Fig. 2. We prove (in lemma 4 in MM Section “Proving Convergence of the Autoassociative Recall Algorithm”) that the recall procedure always converges and that the attractors are either fixed points or 2-limit cycles. Joining all operations to a single equation we get:
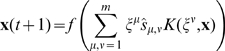
(14)Here by 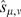 we denote the elements of 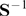. In coordinate form this equation is:
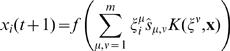
(15)


**Figure 3:**
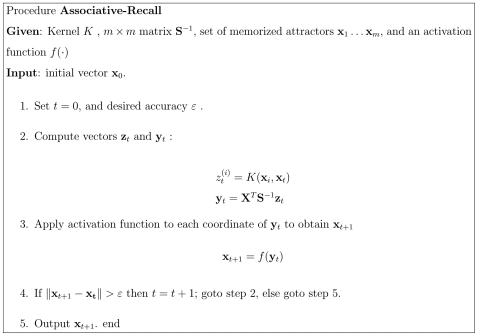
Algorithm of Associative Recall. An iterative procedure converges to an attractor.

The pseudocode of the associative recall is shown in the Fig. 3. As will be shown in the next section, the double nonlinearity of the recall dynamics does not reduce the biological plausibility since the kernel memory can be designed as a layered neural network with only one nonlinear operation per neuron.

In Materials and Methods, Section “Example of the associative recall”, we provide an explicit example of kernel autoassociative memory with 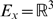 and 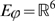. We demonstrate there how a set of five vectors is memorized and how the iterative recall works.

### 2.3 Kernel Associative Memory as a Neural Network

**Figure 4:**
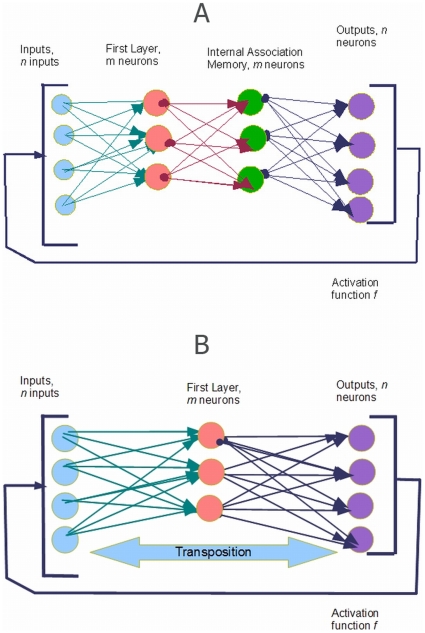
A possible neural-network representation of the Kernel memory. Architecture (a) corresponds to the algorithm of learning and recall as described in the text. In (b) we use an approximation to maximize the capacity and reduce the number of neural units. Middle layer with synaptic matrix 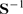 is eliminated, synaptic matrices of the resulting two layers are identical up to transposition. Therefore we have 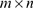 distinct connections (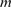 is the number of stored memories and 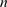 is the input dimension). We can choose a “primary” layer to store the connections, the other one will mirror them. Effective memory capacity of this architecture is 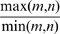.

The autoassociative kernel memory can be directly implemented in a recurrent layered neural network (Fig. 4a): The network has 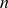 inputs. The first layer has 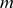 neurons that perform kernel calculations; the 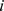-th neuron computes 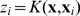. In the special case where the kernel is a radial-basis function 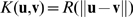 these neurons are the common RBF neurons [23]. The second layer has 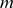 neurons, its weight matrix is 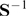. The neurons of the second layer can be either linear or have a generalized sigmoid activation function.

The third layer also has 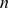 neurons, its weight matrix is 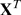. Its activation function can be linear, generalized sigmoid or the even more general sigmoid from Equation (15) above. The network has “one-to-one” feedback connections from the last layer to the inputs. In recall mode it works in discrete time, like Hopfield networks.

**Definition 1** *A monotonic bounded piecewise-differentiable function* 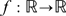 *such that* 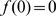, 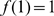, *and* 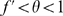 *in certain neighborhoods of 0 and 1 is called generalized sigmoid.*

**Theorem 2** *Suppose that the kernel associative memory has a generalized sigmoid activation function in the second layer. Then the attractors emerging by the iterative recall procedure are either fixed points or 2-cycles.*

Proof appears in Material and Methods Section “Proving Convergence of the Autoassociative Recall Algorithm”.

#### 2.3.1 The Attraction Radius

A key question for any neural network or learning machine is how robust it is in the presence of noise. In attractor networks, the stability of the associative retrieval and the robustness to noise can be measured by the *attraction radius*.

**Definition 2** *Suppose the input to an attractor network belongs to the metric space with distance* 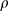. *For an attractor* 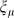 *of the network let* 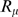 *be the largest positive real number such that if* 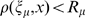 *the dynamics of the associative recall with starting point* 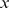 *will converge to* 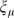. *The value* 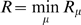 *is called the attraction radius of the network (AR).*

When inputs and memorized patterns belong to a normed vector space, if the additive noise does not exceed the attraction radius in this norm then all memories will be retrieved correctly during the associative recall.

The attraction radius can be estimated in the following special case:

**Theorem 3** *Suppose that a kernel associative memory has identity activation function in the output layer and a generalized sigmoid* 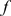 *in the hidden layer. Suppose also the kernel* 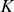 *satisfies the global Lipschitz condition on a certain domain* 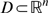, *i.e., there exists* 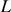 *such that* 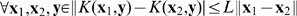. *Then the stored patterns are attractors, and the memory attraction radius is*

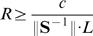

*where* 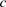 *is a constant that depends only on* 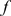.

The proof of this theorem is given in Materials and Methods Sec. Proof of Theorem 3.

**Figure 5:**
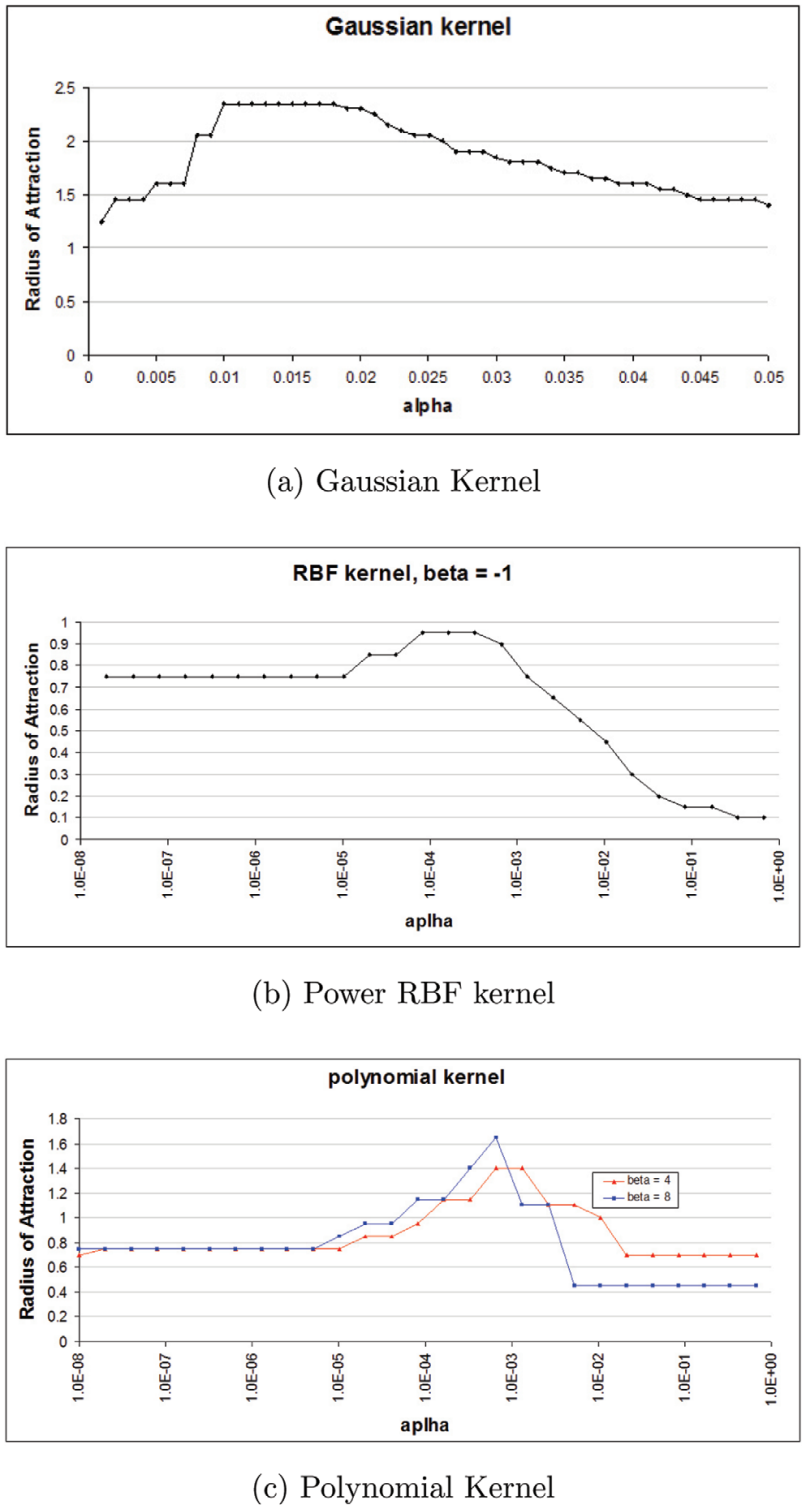
Direct measurement of kernel memory's attraction radius. The dataset of 60 gray-scale 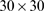 face images was used. They were normalized component wise to the segment 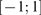. The graphs show dependence of the AR on kernel parameter 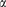 (for fixed 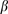) for 3 standard types of kernel: Gaussian 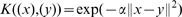 (a); power RBF: 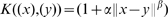, 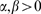 (b), and polynomial: 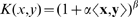, 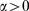, 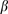 positive integer (c). The AR is relatively large,15–20% of memories' norm, and there exist an optimal value of 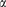 for each kernel.

We further made a series of experiments of direct measurement of attraction radius for a dataset of 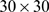 gray-scale face images. Results of this experiment are represented in Fig. 5.

**Figure 6:**
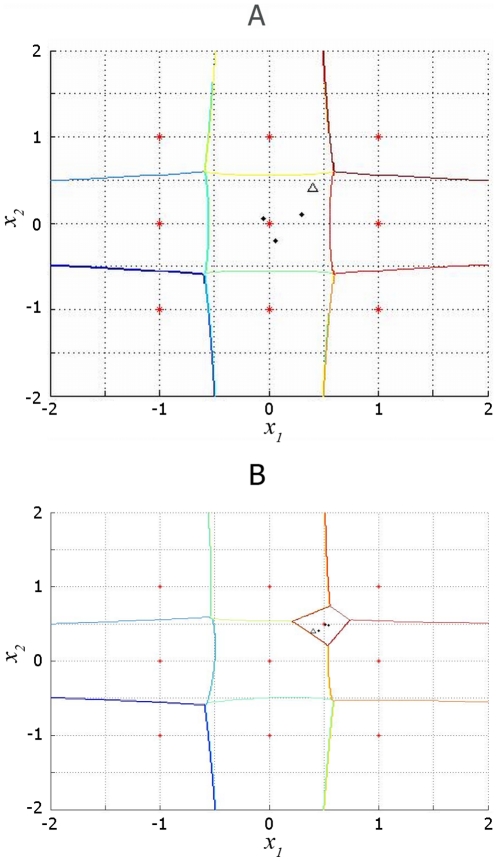
Example of an explicit insertion of an attractor. (a): 9 attractors in 2D (red stars), attraction basins are bounded by Voronoi polygons. (b): A tenth attractor 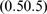 is added. Trajectories of the associative recall dynamics starting at (0.4, 0.4) (triangle) are shown in black dots. The destination attractor for this initial condition has been changed since the new attractor was stored.

**Remark 3** We have also proven that under the conditions of Theorem 3 all stored patterns are attractors. Yet, the memory was not proven to be free from spurious equilibria. However, spurious attractors were never observed in numerical experiments. The typical situation is that all the input domain is divided into attraction basins of the memorized vectors. The basins look like Voronoi polyhedra as depicted in Fig. 6.

#### 2.3.2 Bounded Synaptic Weight Range

There is a connection between a bound on the values of the synapses (see [45]) and the kernel function defining the network.

**Proposition 1** *Let the kernel memory have input data such that every two inputs* 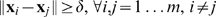 *and the piece-wise Mercer kernel* 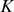 *with this* 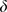 *and certain* 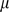. *Then the synapses defined by matrix* 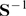 *are bounded and the following bound holds:*

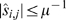



**Proof.** From the proof of Lemma 3.2 in Material and Methods we know that 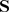 could be written as 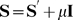, where 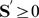 is a positive semidefinite matrix. By linear algebra we have 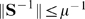. Finally 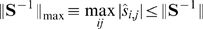 by matrix norm equivalence in finite-dimensional space.

#### 2.3.3 Maximizing Capacity

The kernel associative memory works as a symmetric network in an abstract feature space is used only in implicit way. Any implementation of the kernel associative memory with neural computational units requires a recurrent layered structure (Fig. 4). We can maximize the network capacity by using the approximation 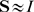. This approximation is suitable if the stored patterns are sufficiently distant in the kernel view, see Remark 5. With this approximation one can save 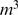 connections without significant loss of association quality by eliminating the middle layer in Fig 4a and the other two layers will have weight matrices 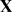 and 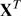, identical with respect to transposition; see Fig. 4b. So, to store 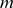 vectors of 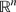 we would need 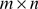 real numbers only (lossless coding).

The definition of memory capacity and connections/neurons ratio now leads to
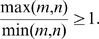



**Remark 4** The approximation 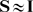 is suitable if the stored patterns are almost orthogonal in the feature space. For localized kernels (e.g. RBF) this means that the patterns are distant enough from each other (comparing to the characteristic scale of the kernel). Because the condition of orthogonality is applied in the feature space, not in the input space, this condition does not imply any relative size of 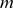 versus 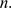


With this optimization the kernel-memory network can be made arbitrarily sparse by choosing a sparse kernel, i.e., a kernel that explicitly depends only on a (small) portion of the coordinates of its argument vectors. The non-zero weights will correspond to the inputs that the sparse kernel depends on. The attractors will have a sparse structure analogous to the kernel as well. If our goal is to memorize arbitrary dense data we can still use the sparse network as long as encoding and decoding layers are externally added to it.

### 2.4 Flexibility of the Memory

#### 2.4.1 Flexibility in the Attractor Space

The kernel associative memory can be made capable of adding and removing attractors explicitly.

To add a new attractor to the network we create a new neuron in the **S** matrix layer. The dimension of matrix 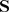 is increased from 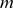 to 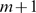. To do so we compute 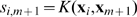 and we update the inverse 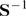 efficiently using the linear-algebra identity [46]:
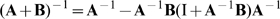
(16)where 
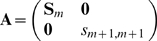
 and
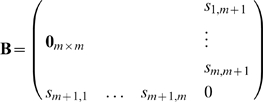
Calculation using (16) takes 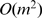 operations since 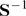 is already known.

Similarly one can delete an attractor by reducing the dimension of **S**. Here
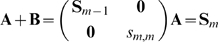


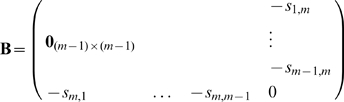
To receive 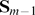, the last column and last row from matrix 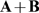 will be removed.

**Figure 7:**
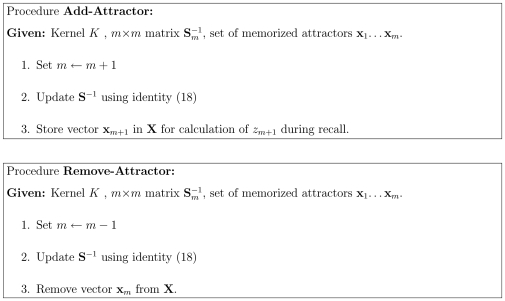
Procedures of adding and removing attractors in kernel autoassociative memory.

This results in the two algorithms of Fig. 7.

**Remark 5** In the case where 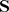 is approximately a diagonal matrix, its inverse can be calculated by the approximation 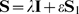 for small 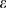, and 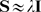, which does not change during updates.

**Remark 6** The procedure Add-Attractor (see Fig. 7) is local in time. To store a new pattern in the memory we only have to know the new attractor and the current connection matrix 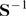.

In Fig. 6 we display an example of adding an attractor.

#### 2.4.2 Flexibility in Input and Feature Spaces

External inputs may come with more or fewer features than previously, causing the input dimensionality to change with time. We propose a mechanism that enables the network to handle such heterogeneity of dimension with no need to relearn the previously learned inputs.

Assume that the current dimension in the input space consists of the “initial dimension” 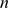 and “new” 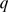 dimensions; denote this as 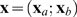. We will allow the change of dimensionality by changing the kernel itself: from the kernel 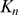 that considers the first 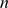 dimensions to kernel 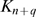 that depends on all dimensions.

**Figure 8:**
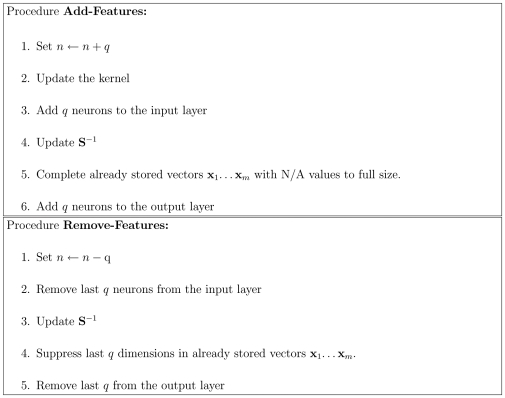
Algorithms assuring flexibility in the input dimension.

The change of kernel requires the recalculation of 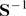. However, this need not require 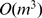 operations if we constrain to kernels that can be written in an additive form:
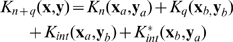
(17)where 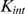 describes the interaction of 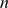 and 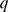. An explicit kernel with this property is the polynomial kernel (see Section “Variable Kernel and Dimensionality”). Algorithms for dimensionality control appear in Fig. 8. An example is given in Example 3 in the next section.

We also prove Lemma 4 in Materials and Methods Section “Variable Kernel and Dimensionality”, stating that a small alteration to the kernels enables changing input dimensionality without loosing previously learnt attractors.

### 2.5 Memory Consolidation and Clustering

The memory system with its loading algorithm enables consolidation of inputs into clusters using the competitive learning method. Suppose we have a learning sample of 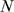 vectors 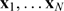 and 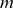 clusters have to be created. Random vectors initiate the 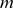 attractors. When a new input is provided, the recall procedure is performed and the attractor of convergence 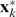 is updated by 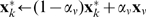. Parameter sequence 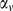 is selected in order to provide better convergence of attractors: for instance, we can take 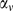 such that 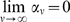 but 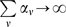. This step is repeated until all attractors stabilize.

We tested the consolidation algorithm using the MNIST database of handwritten digits [47]. The data consists of ten different classes of grayscale images (from ‘0’ to ‘9’, each of 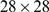 pixels in size) together with their class labels.

**Experiment 1:** *Clustering with the Kernel Memory*. The goal of this experiment is to demonstrate performance of memory clustering. For this purpose memory was trained on the learning sample in order to form attractors. Then attractors were assigned to classes, and classification rate was measured on an independent test sample.

For the MNIST data we used principal-component (PC) preprocessing. We took the first 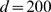 PCs which contain 96.77% of the variance. The learning sample contained 10000 digits, 1000 from each class. The kernel was chosen in the form:
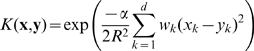
(18)where 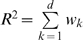, and 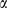 is a parameter. This is a Gaussian kernel depending on weighted metric. Weights were chosen as:
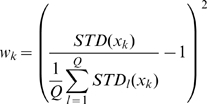
(19)


We also tried the formula:
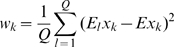
(20)where 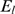 and 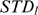 are expectation and standard deviation over the 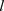-th class, and 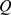 is the quantity of classes. However formula (19) gave better results.

**Figure 9:**
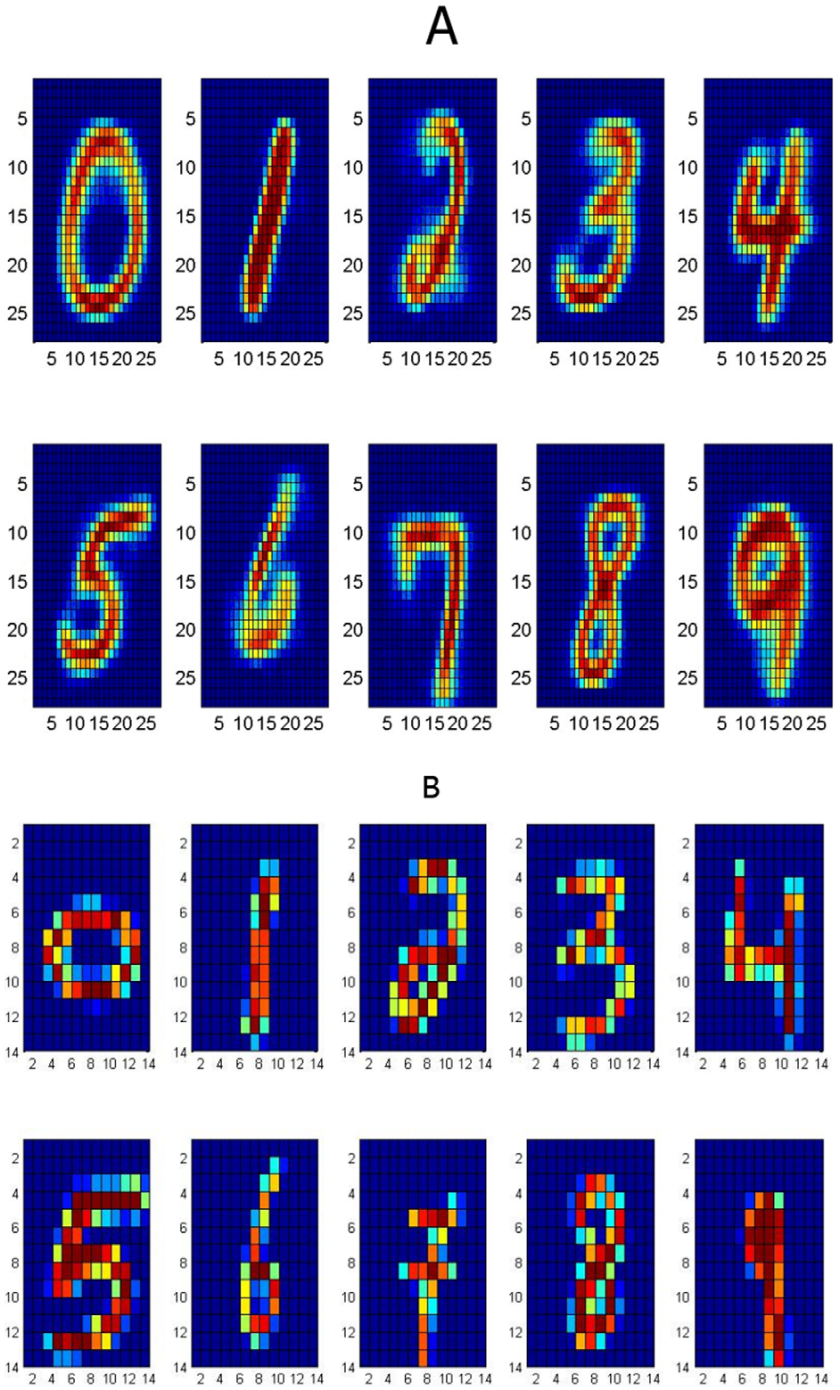
The MNIST experiment. (a): Ten typical attractors, one for each class out of 100 attractors. (b): An example of 10 downscaled digits. Each digit in (b) is a 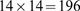-dimensional vector. Experiment 2 demonstrates work of the algorithm of adding features. we started learning with 10000 downscaled images. To continue training we used another 10000 images, and we had to add 784–196 = 588 features.

Because of the complexity of the MNIST data, we chose to have multiple clusters per class. Table 1 summarizes the classification rates for different amounts of attractors in the memory. The classification is slightly superior to other unsupervised clustering techniques (even that the goal of the memory system is not directly in clustering). The number of memory attractors required for good clustering is also smaller than other techniques, e.g. [48]. Figure 9.a) provides an example of typical memory attractors of each class.

**Table 1:** Experiment 3.

**Attractors per class**	1	10	20	50	100
**Classification rate, %**	52.4	80.4	82.2	87.8	91.1
**Classification rate**, 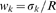 , %	34.5	74.48	82.3	89.09	91.38

MNIST digits clustering. Classification rate vs. number of concepts.

We also made a series of experiments with 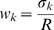; 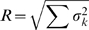, where 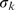 is an STD of 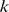-th principal component. This weighting does not depend on class labels in any way. We can see (last row of table 1) that results are poor for small number of attractors per class, but for higher number of attractors classification rate is even better.

**Experiment 2.** *Clustering under changing input dimensionality*. This experiment demonstrates clustering while input dimensionality increases and the kernel is being changed. For this purpose, the resolution of the original images was reduced twice, to 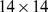 (Fig. 9). Then the images were passed through a linear transformation in order to use the kernel (19). The memory was trained on 10,000 such digit images, forming 100 attractors. The recognition quality obtained was 76.4%. Then the kernel was extended in order to work with the original size 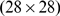, and another 10,000 digits were added, now in full-size. This second session of learning started from the previous set of attractors, without retraining. The final classification rate was enhanced to 85.4%.

**Experiment 3.** *Explicit example of adding input dimension*. Consider the 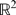 data where points lie on two Archimedes' spirals:
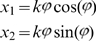
(21)and
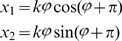
(22)We chose angle range 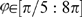 for both classes. The initial kernel was 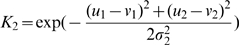. Then we add 3-d coordinate 
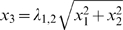
, where 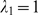 and 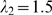 for first and second class. For 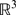 the additive kernel will be 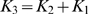, where 
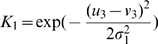
, an interaction term is not necessary in this example. We took 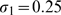 and 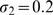. At first, the network was loaded with the 40 data points in 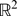. Each point was labeled and a classification was executed. The recognition quality on an independent test sample was 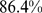. Then the training was continued with the additional 40 inputs in 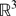 and the final classification rate increased to 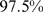.

### 2.6 Synaptic Plasticity and Memory Reconsolidation

Reconsolidation is a storage process distinct from the one time loading by consolidation. It serves to maintain, strengthen and modify existing memories shortly after their retrieval [49]. Being a key process in learning and adaptive knowledge, problems in reconsolidation have been implicated in disorders such as Post Traumatic Stress disorder (PTSD), Obsessive Compulsive disorder (OCD), and even addiction. Part of the recent growing interest in the reconsolidation process is the hope that controlling it may assist in psychiatric disorders such as PTSD [50] or in permanent extinction of fears [51].

#### 2.6.1 Current Model of Reconsolidation in Hopfield Networks

A model of reconsolidation was introduced in [19]. It contains a learning mechanism that involves novelty-facilitated modifications, accentuating synaptic changes proportionally to the difference between network input and stored memories. The formula updating the weight matrix 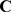 is based on the Hebbian rule:
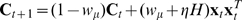
(23)


Here 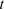 is the time of the reconsolidation process, 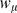 is a weight parameter defining learning rate, 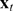 is the current input stimulus, 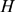 is a Hamming distance from 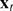 to the set of network's attractors, and 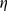 is the sensitivity to the novelty of stimuli. This formula differs from the original Hebbian rule by having both weight decay and Hamming-distance terms affecting the learning.

The model predicts that memory representations should be sensitive to learning order.

#### 2.6.2 Our Reconsolidation Algorithm

In the case of Hebbian learning, the network's synaptic matrix is composed of a linear space. In our kernel associative memory, on the other hand, the corresponding space is no longer linear but rather is a Riemannian manifold, see Materials and Methods Section 3.7. Additions and multiplications by a scalar are not defined in this space and thus formula (23) cannot no longer be applied.

**Figure 10:**
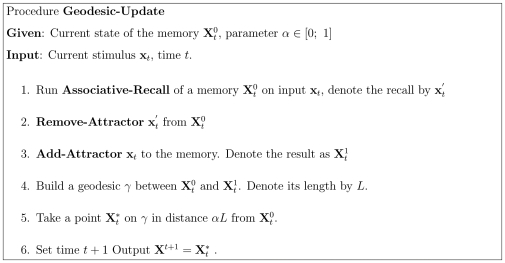
Algorithm of Geodesic Update.

To remedy the situation we define a Riemannian distance (see Material and Methods Section 3.7) and a geodesics which enables the memory to change gradually as new but close stimuli arrive [52]: a point on a geodesic between 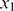 and 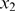 that divides the path in ratio 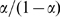 is a generalization of the convex combination 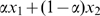. Suppose, initially we have a memory 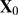 that contains 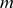 attractors 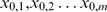. Then we obtain 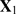 by replacing one attractor by a new stimulus: 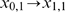. The distance between 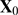 and 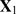 can be thought of as a measure of “surprise” that the memory experience when meets new stimuli. To track the changes, the memory moves slightly on the manifold from 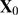 to 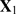. See algorithm in Fig. 10.

#### 2.6.3 Numerical Experiments

We exemplify the power enabled to us by the reconsolidation with the following experiments.

**Experiment 4**. *Morphed faces*. The goal of this experiment is both to show the performance of the reconsolidation process we describe on large-scale data and to compare its properties with the recent psychological study [35].

Morphed faces were created by Joshua Goh at the University of Illinois. The faces were obtained from the Productive Aging Lab Face Database [53] and other volunteers (All the face images used in our research were taken from the Productive Aging Lab's Face Database, Morphed face dataset. This dataset is freely accessible from https://pal.utdallas.edu/facedb/request/index/Morph). They contain a mix of young, old, Asian, Western, male, and female faces. They are gray-scale with luminance histogram equated. Faces were morphed using the software Sqirlz morph. Original size of all images was 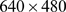. Useful area falls in the rectangle 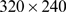, images were cropped to this size before entering to the network. The database contains 150 morph sequences, each of them consists of 100 images.

**Figure 11:**
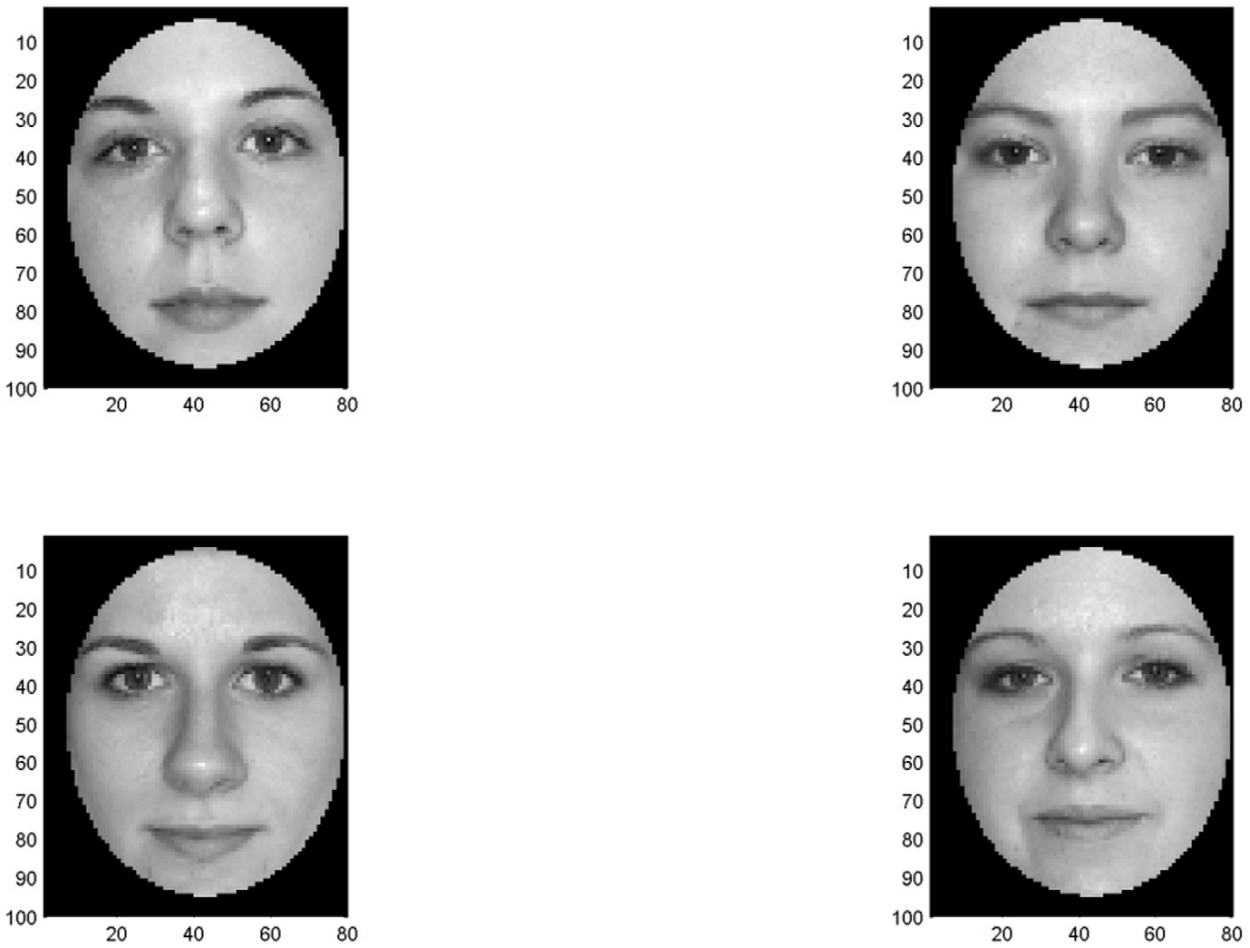
Example of attractors during face reconsolidation.

In our simulations we created a network with 16 attractors representing 16 different faces; it had 76800 input and output neurons, and two middle layers of 16 neurons each. Four arbitrarily selected network's attractors are depicted in Fig. 11. A Gaussian kernel was chosen in order to simplify calculations with large scale data.

Attractors were initialized with first images from 16 arbitrarily selected morph sequences. When the learning order followed image order in the morphing sequence, attractors changed gradually and consistently. The ability to recognize the initial set of images gradually decreased when attractors tended to the final set. In case of random learning order attractors quickly became senseless, and the network was not able to distinguish faces.

This experiment generalizes the result shown in [19] but is done on real images demonstrating the efficiency of the reconsolidation process in kernel memories for high dimension and multi-scale data. In accordance with [35], the formation of “good” dynamic attractors occurred only when morphed faces were presented in order of increasing distance from the source image. Also, as shared also with [34], [19]: the magnitude of the synaptic update due to exposure to a stimulus depends not only on the current stimulus (as in Hebbian learning) but also on the previous experience, captured by the existing memory representation.

**Experiment 5**. *Tracking Head Movement*. This example focuses on rotating head images for reconsolidation based on the VidTIMIT dataset [54], and it demonstrates our algorithm on a more applied example of faces and computer vision. The VidTIMIT dataset is comprised of video and corresponding audio recordings of 43 people. The recordings include head rotation sequences. The recording was done in an office environment using a broadcast quality digital video camera. The video of each person is stored as a numbered sequence of JPEG images with a resolution of 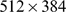 pixels.

**Figure 12:**
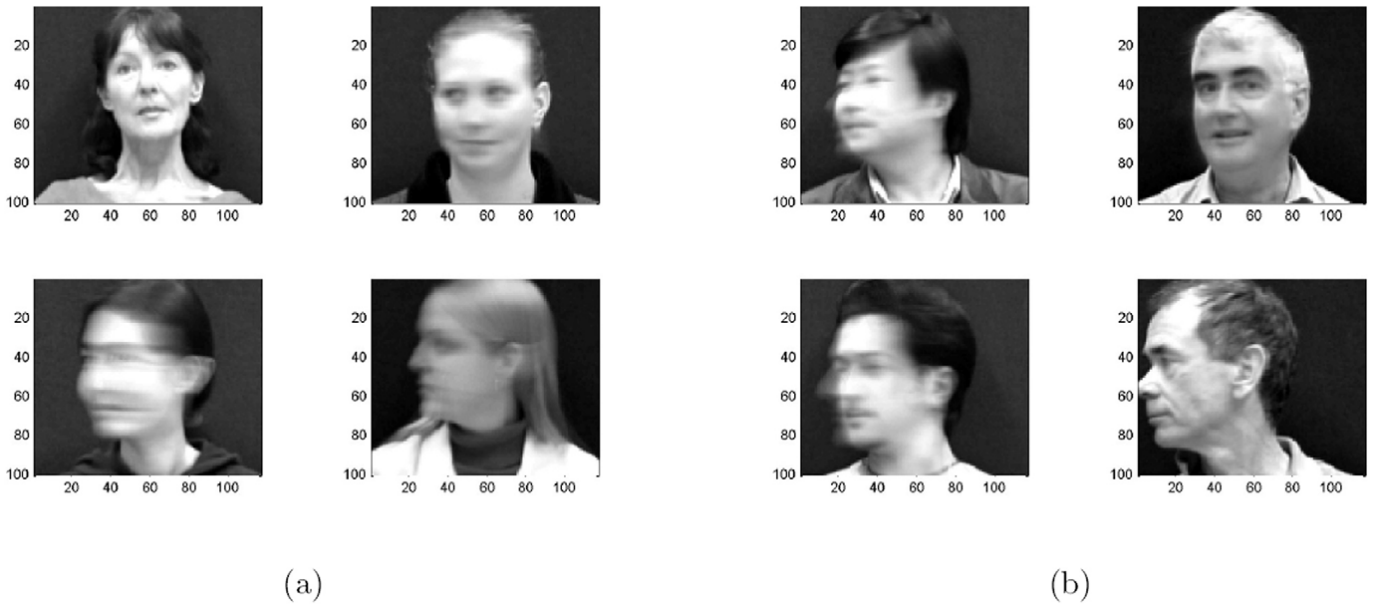
Tracking rotating heads via reconsolidation. Memory attractors are blurred when motion is quick.

The ability to track and recognize faces was tested on the sets of 15 last frames from each sequence. Example of attractors during the reconsolidation in this experiments is depicted in the Fig. 12. With reconsolidation and ordered stimuli the obtained recognition rate was 95.2%. If inputs were shuffled randomly, attractors got messy after 30–50 updates, and the network did not demonstrate significant recognition ability.

**Experiment 6**. *Tracking The Patriot Missiles*. The following experiment takes the reconsolidation model into a practical technology that follows trajectories in real time in complex dynamic environments.

We analyzed videos of Patriot missile launches with resolution 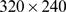, originally in RGB color, and transformed them to grayscale. The memory was loaded with vector composed of two 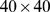-pixel regions (windows) around the missile taken from two consequent frames and a two-dimensional shift vector indicating how the missile center has moved between these frames. Optimal number of attractors was found to be 16–20.

**Figure 13:**
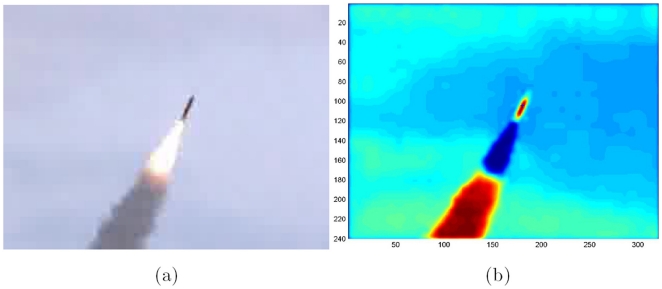
Patriot missile example. Original frame (a) and a processed frame with tracking marker (b).

Using memory reconsolidation algorithm we were able to calculate velocity vector every time, and therefore track the missile with great precision, with only average error of 5.2 pixels (see Fig. 13).

### 2.7 Conclusions

We have proposed a novel framework of kernel associative memory as an attractor neural network with a high degree of flexibility. It has no explicit limitation, either on the number of stored concepts or on the underlying metric for association, since the metric is based on kernel functions. Kernels can be slightly changed as needed during memory loading without damaging existing memories. Also due to the kernel properties, the input dimension does not have to be fixed. Unlike most other associative memories our model can both store real -valued patterns and allow for analogous attractor-based dynamic associative recall.

We endowed our memory with a set of algorithms that insure flexibility, enabling it to add/delete attractors as well as features (dimensions) without need to retrain the network. Current implementation of our memory is based on a simple competitive clustering algorithm and consolidates memories in a spirit similar to the localist attractor networks [43]. We have experimentally tested the memory algorithms on the MNIST database of handwritten digits, a common benchmark for learning machines. The obtained clustering rate for this database (91.2%) is slightly better than the best known result for unsupervised learning algorithms on this benchmark. The model further allows the process of reconsolidation after memory is stored when retrieval by similar patterns is activated. We demonstrated the properties of reconsolidation on gray scale large image faces in morphing experiments. Based on the theoretical and experimental research made in the present paper we conclude that the proposed kernel associative memory is promising both as a biological model and a computational method.

## Materials and Methods

### 3.1 Piece-wise Mercer Kernels

The classical Kernels 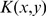 introduced by Vapnik to the field of Machine Learning had the Mercer condition. That is, for all square integrable functions 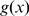 the kernel satisfied:
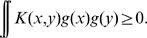
(24)The Mercer theorem states that if 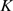 satisfies the Mercer condition there exists a Hilbert space 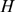 with basis 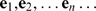 and a function 
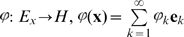
, where 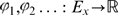, such that
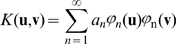
(25)and all 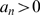. That is, 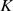 is a scalar product of 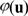 and 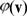


General Mercer kernels are not sufficient for creating the associative memory since our kernel memories require that all attractors are linearly independent in the feature space, and some Mercer kernels do not assure it, such as the basic scalar-product kernel 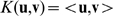. As shown in Lemma 2, linear independence of attractors in the feature space is needed to provide correct association in our system. The piece-wise Mercer kernels as we define next have this desired property.

**Definition 3** *A kernel* 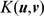 *is said to satisfy the piece-wise Mercer condition if there exist* 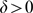, 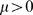, *and a Mercer kernel* 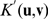 *such that:*

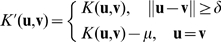
(26)
*and*

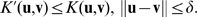
(27)


The piece-wise Mercer kernel is an extension of strictly positive definite (SPD) kernels. These kernels have some internal property of regularization. For usual Mercer kernels, e.g. common scalar product there are still situations when Gram matrix of certain vector set in the feature space will be degenerate. In contrast to this, the 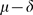 piece-wise Mercer kernel can guarantee that for any finite set of vectors their Gramian will be full-rank and even greater than 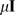.

**Lemma 1** *If* 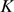 *is a piece-wise Mercer Kernel, it also satisfies the Mercer condition.*

**Proof.** Indeed, for all square integrable functions 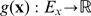, the Mercer condition and inequality (26–27) give:
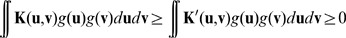
and the Mercer condition for 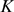 is fulfilled.

**Remark 7** There is following relation between our definition of piece-wise Mercer kernel and standard notion of strictly positive definite (SPD) kernel (which is formulated by replacing “
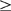
” with “
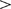
” in (24): any piece-wise Mercer kernel is also SPD and for any continuous SPD kernel 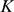 there exist certain 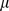 and 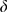 such that 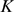 is 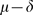 piece-wise Mercer.

**Example 1** *We demonstrate how to build a piece-wise Mercer kernel that would fit a given sample to be loaded in memory. Consider Gaussian Kernel* 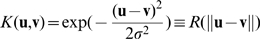. *We can construct the kernel:*

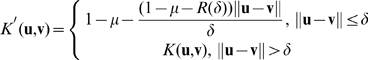
(28)
*The kernel* 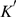 *is constructed in that way that it is continuous and convex as a function of* 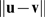 *for any* 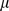 *and* 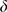 *such that* 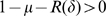. *According to the Polya criterion (see,* [*37*] *, sec. 10.8.4) we get that* 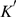 *fulfills the Mercer condition. This shows that Gaussian kernel* 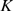 *is a piece-wise Mercer kernel.*

### 3.2 Correctness of Association

**Lemma 2** *Suppose kernel* 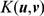 *satisfies the piece-wise Mercer condition for certain* 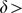 *0 and there are input vectors* 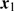 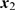 *…* 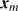 *such that* 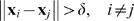. *Then there exists a Hilbert space* 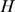 *and a nonlinear transformation* 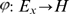 *such that*

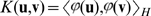

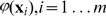
 *are linearly independent.*

**Proof.** Since 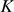 is a Mercer kernel according to lemma 1, statement 1 is true by the Mercer theorem.

To prove 2) note that by definition 1 there exists a kernel 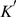 that satisfies the inequality (26). 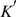 is a Mercer kernel, and hence matrix 
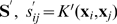
 would be non-negative definite. By our choice of 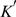, 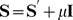 is strictly positive then invertible, as a sum of a positive-semidefinite and positive scalar matrices. That means that 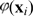 are linearly independent in 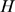, and we are done.

We use the following facts from linear algebra:

- *Sum of a positive semidefinite matrix and positive scalar matrix is a strictly positive (symmetric) matrix*
- *If matrix of pairwise scalar products of a finite set of vectors in Hilbert space is strictly positive the vectors are linearly independent.*

**Lemma 3** *For any given finite learning sample* 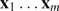 *of distinct vectors there exists a Kernel* 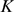, *Hilbert space* 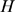, *and a nonlinear transformation* 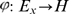 *such that*

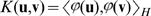

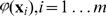
 *are linearly independent.*

**Proof.** Take 
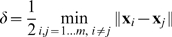
. Let us take Gaussian Kernel from Exapmple 1 with 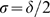. As we have shown above in the Example 1, it will satisfy piece-wise Mercer condition with 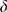 and any 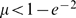. Then we apply Lemma 2 to 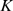 and we are done.

Next we provide the proof of Theorem 1.

**Proof.** Let 
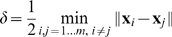
. Pick a kernel that satisfies piece-wise Mercer condition with this 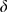 and certain 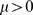. Existence of at least one such a kernel for every 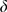 is guaranteed by Example 1 (see MM) that shows how to construct piece-wise Gaussian kernels. Then, the conditions of Lemma 2 are fulfilled by kernel 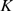 and input set 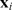. Therefore, 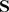, is invertible, 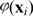 are independent in the feature space, and association (9) — (11) is well defined.

### 3.3 Example of the associative recall

**Example 2** ***Autoassociative memory*** *. Let* 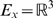, *and* 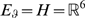. *Take,*

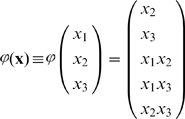

*The kernel will be*

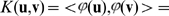


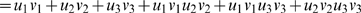

*Take sigmoid activation function:* 
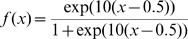


*Suppose we have to memorize the following* 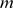 *= 5 vectors:*

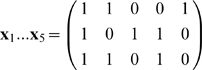

*If we apply* 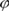 *to this set we have*

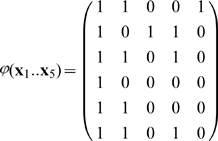

*Vectors became linearly independent but their dimension has inflated. The matrix* ***S*** *is:*

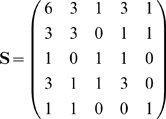

*det(* ***S*** *) = 4, we can compute its inverse:*

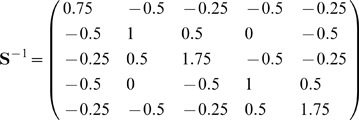

*Suppose the input vector for recall is* 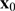 *= (0.22, 0.75, 0.8)* 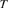.

***First iteration*** *. Starting from* 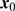 *compute* 
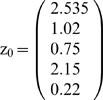
 *then* 
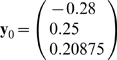
, *and* 
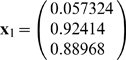
.

*Second iteration leads to* 
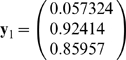
 *and* 
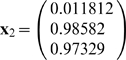
. *So, the process converges to attractor (0,1,1).*

### 3.4 Proving Convergence of the Autoassociative Recall Algorithm

**Lemma 4** *Suppose we have an autoassociative memory with kernel* 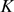, *stored vectors* 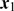 *…* 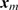 *forming columns of matrix* 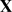, *and a matrix* 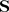 *with elements:*

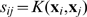

*The dynamical system corresponding to the associative recall is:*

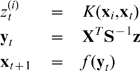
(29)
*Suppose kernel* 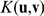 *is continuous, and it satisfies the piece-wise Mercer condition for a certain* 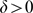 *such that the stored vectors* 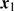 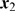 *…* 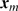 *satisfy* 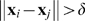 *for* 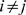. *Then attractors of the dynamical system (29) are either fixed points or 2-cycles.*

**Proof:**

We will prove that 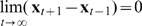. For this we construct an energy function in a way that is analogous to Hopfield-like networks:
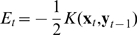
(30)


**Step 1. Show that the energy has lower bound.** Because 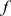 is bounded, the closure of the co domain of 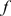 in (29) is a certain compact set 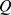. So, 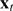 will remain in 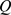 for all 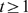. The energy (30) is bounded over 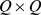 as a continuous function over a compact set.

**Step 2. Show that there exists a projective self-conjugated operator** 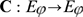 such that 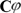
(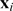) = 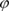
(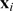), *i = 1…m*. By theorem 1 there exists a feature space 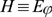 such that the kernel gives a scalar product in this space.

For every finite set of vectors 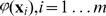 in 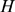 we can construct a projective operator 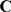 that projects to the subspace spanned with 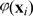. Indeed, applying Gram-Schmidt **orthogonalization** to 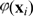 (see, e.g. [55]) we can build an orthonormal set (basis) of vectors 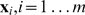.

Then define an operator as follows:
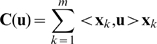
This 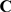 is projective, and it projects to the finite-dimensional subspace 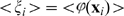. (Here by 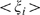 we denote a subspace spanned with all 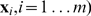.

**Step 3. Show that energy decreases monotonically every 2 steps.**

Applying properties of the scalar product in 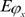 and symmetry of 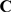 we get:
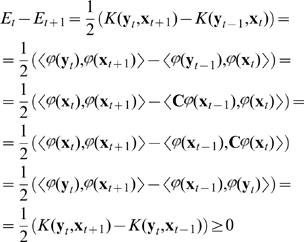
(31)Because 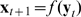, 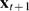 is closer to 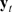 than any other point on the trajectory, and the kernel is monotonic with respect to distance between 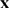 and 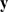, the expression (31) will be non-negative. Moreover, is 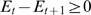; it is zero if and only if a fixed point is reached.

**Step 4. Show that the total amount of energy decrease is finite if and only if sequences** 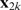 **and** 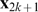 **converge.** Suppose the energy lower bound is 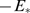.
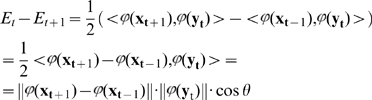
(32)So, there exists 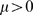 such that 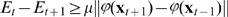.

Then 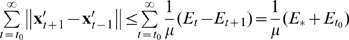. The sum on the left hand of this equation will be finite if and only if sequences 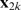 and 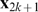 converge, and we are done.

**Remark 8** *By Theorem 3 and Remark 2 we have proven that (all) stored patterns are attractors. This means that* 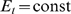 *if* 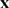 *equals one of stored vectors.*

We next prove Theorem 2.

**Proof.** The proof is analogous to the proof of lemma 4. Note that if the 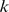-th attractor is reached 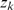 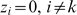. Activation function in the form of generalized sigmoid brings 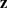 closer to an attractor. So, where in the proof of lemma 4 the linear activation function is replaced with a generalized sigmoid, convergence to an attractor can only be fasted reached.

### 3.5 Variable Kernel and Dimensionality

For polynomial kernel of degree 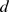 we have:
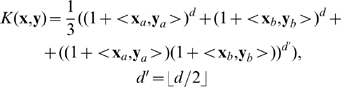
(33)For such decomposed kernels, the matrix 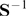 can be efficiently (O(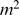)) updated using formula (16).

**Lemma 5** *If the kernel* 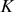 *is a linear combination of basis functions, there exists an additive kernel* 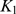 *having the same feature space* 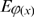.

Having the same feature space is important because it leads to identical behavior of two kernel memories with these to kernels. We note that as before, if 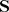 is an approximately diagonal matrix, the inverse 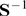 can be estimated efficiently. A diagonal matrix appears for example in the Radial-Basis-Function-like kernels. In this situation the approximations are given by 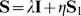.

#### 3.5.1 Memory Stability with Changing Kernels

Suppose all vectors submitted for learning and recall belong to a certain compact set 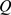. Let us define the norm for kernels:
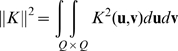



**Proposition 2** *For given space* 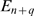 *and a compact set* 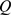 *in it there exists a constant* 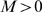 *such that if* 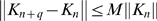 *for any set of vectors* 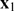 *…* 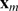 *stored in the memory with* 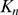 *these vectors remain in attraction basins of corresponding attractors for the memory with kernel* 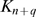 *whose attractors expand* 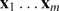 *to dimension* 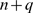.

**Proof.** The proof directly follows from the norm definition and direct estimation of attractor shift when the kernel is changed.

### 3.6 Proof of Theorem 3

**Lemma 6** *Let* 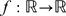 *be a generalized sigmoid. Suppose* 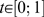 *and variable* 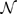 *takes two values 0 and 1. Then there exist constants* 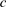 *and* 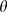 *in* 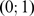 *such that if* 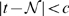, 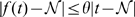


**Proof.** By definition 1 there exist 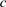 and 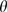 in 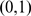 such that 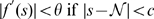. Estimate:
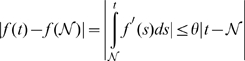
Here we prove Theorem 3.

**Proof.** Select one of stored patterns 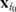 and denote it as 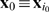. Consider the vector 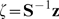. If 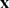 is equal to, one of stored vectors, corresponding vector 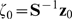 has all zero coordinates except 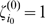, equivalently 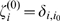.

Estimate the norm 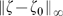.
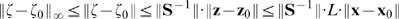
If 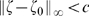, 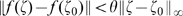 according to lemma 6. So, if
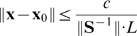
iterations of the recall will make 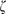 converge to 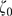 with exponential velocity, that immediately implies convergence of 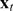 to 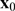, and we are done.

### 3.7 Reconsolidation and Riemannian Distance

#### 3.7.1 Riemannian Metric

Riemannian manifold is a smooth, at least twice differentiable manifold (see [52], [56], or any textbook on Riemannian Geometry), which has a scalar product at every point. The scalar product, or Riemannian Metric, is defined in a tangent space of a point as a positive quadratic form.

Riemannian metrics enables to measure curve length and introduce geodesics: trajectories of a free particle attached to the manifold. Between every two points there exist at least one geodesic that have minimal length among all curves joining these two points. Length of this geodesic gives Riemannian distance over the manifold.

There is following Riemannian distance between two kernel associative memories:
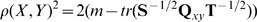
(34)Here 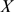 and 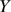 are two memories with the same 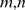, and 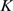. They have 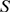-matrices 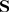 and 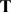 respectively and 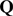 is the “cross-matrix”: 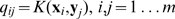.

#### 3.7.2 Geodesic Update

To find a point analogous to convex combination of 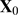 and 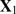 we build a geodesic 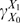 joining these to points, and take a point 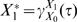. Here 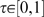 is a step parameter related to size of a shift during each update. For 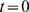 we stay at 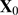, for 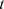 = 1 the point is changed to 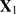. Repeatedly, we track from 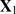 to 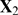 when a stimulus 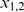 appears, etc.

The algorithm of memory update using geodesics uses the property of kernel memory that an arbitrary attractor can be added or removed to the network in 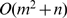 operations with no impact to all other attractors.
